# COVID or Not COVID? A Great Mimicker Behind the Smoke Screen

**DOI:** 10.7759/cureus.19480

**Published:** 2021-11-11

**Authors:** Malek Ayoub, Megan Quamme, Abdul-Rahman K Abdel-Reheem, Poe Lwin

**Affiliations:** 1 Department of Internal Medicine, Medical College of Wisconsin, Wauwatosa, USA

**Keywords:** e-cigarette and vaping product use associated lung injury (evali), tetrahydrocannabinol (thc), covid 19, dyspnea, vaping and covid-19, vaping

## Abstract

Vaping is becoming increasingly popular as an alternative to cigarettes. However, vaping does not come without risks; electronic cigarette (e-cigarette) and vaping-associated lung injury (EVALI) is one of the most severe consequences. Coronavirus disease 2019 (COVID-19) and bacterial pneumonia cases often present with almost identical features. We present a case of a young man who presented with pneumonia that was initially thought to be related to COVID-19 infection but later diagnosed as EVALI. Clinicians should have a high suspicion of EVALI in patients who present with hypoxemia and negative infectious workup, particularly during the COVID-19 era. Administration of corticosteroids has shown remarkable efficacy in improving hypoxemia; however, many patients may have chronic lung injury and may require oxygen long-term. Cases of EVALI should continue to be reported and followed up long term for monitoring disease outcomes.

## Introduction

A number of cases of e-cigarette or vaping-associated lung injury (EVALI) were reported in 2020. To date, more than 2,807 hospitalized EVALI cases have been reported to the Center for Disease Control (CDC) from 50 states, and 68 deaths have been confirmed [1]. We present a case of a young patient who presented with pneumonia that was thought to be coronavirus disease 2019 (COVID-19) at presentation but was later diagnosed to be EVALI. Reporting and creating awareness is pivotal for improving EVALI associated morbidity and mortality. 

## Case presentation

Prior to COVID-19 vaccination becoming widely available, a previously healthy, 20-year-old Caucasian male with no significant past medical history presented with two weeks history of progressive dyspnea. About four days prior to presentation, he was evaluated at the emergency department with dyspnea, nonproductive cough, malaise, emesis, diarrhea, intermittent fever with a temperature max of 103°F, and found to have leukocytosis of 20,000 and negative COVID-19 test. Chest X-ray (CXR) showed bilateral interstitial patchy opacities and he was prescribed a five-day course of azithromycin for possible atypical pneumonia. He failed to improve after four days of treatment with azithromycin. He presented with worsening hypoxemia associated with fever, cough, emesis, and diarrhea.

**Figure 1:**
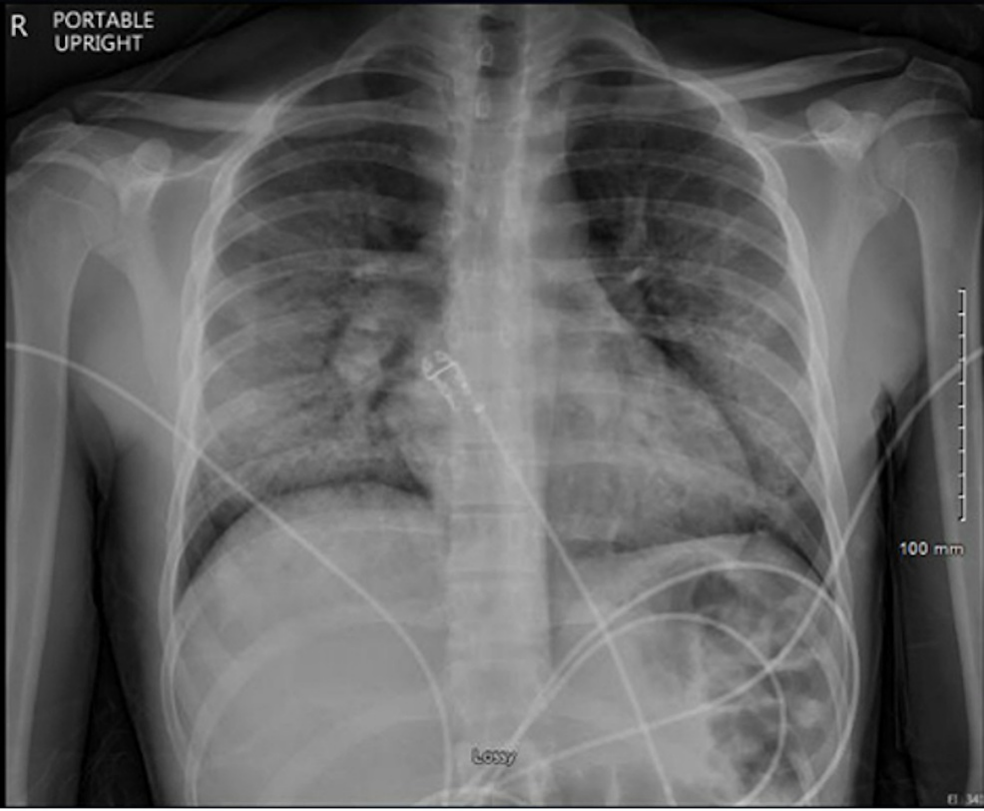
Chest X-ray at the time of admission showing bilateral patchy infiltrates.

On presentation, vitals showed temperature of 100.8°F, heart rate 99, blood pressure 129/81, respiratory rate 40, SpO2 85% on room air which improved to >90% on 4L nasal cannula. The patient was not in respiratory distress and a physical exam showed crackles over right lung fields and left lingula with expiratory wheezes in the same distribution. Labs were significant for leukocytosis (18x10^3/uL) with eosinophilia (7%). CXR demonstrated diffuse bilateral airspace and interstitial opacity concerning for infection or inflammatory process including COVID-19 (Figure 1). Azithromycin was continued and ceftriaxone was initiated for possible community-acquired pneumonia (CAP).

The patient was placed in isolation for CXR findings, as mentioned above, which was concerning for COVID infection. Repeat testing for COVID came back negative. Further workup was significant for elevated ferritin 1,258 ng/mL, lactate dehydrogenase (LDH) 734 units/L, c-reactive protein (CRP) 27.20 mg/dL, procalcitonin 0.58 ng/mL suggestive of possible false-negative COVID-19 infection vs. CAP. Eosinophilic pneumonia vs. allergic bronchopulmonary aspergillosis (ABPA) was considered for eosinophilia and it was noted on initial complete blood count (CBC); further workup with IgE was unremarkable with 9.7 U/ml. Extensive infectious work up with influenza, mycoplasma, chlamydia nucleic acid amplification test (NAAT), sputum culture, urine antigen testing for legionella, Histoplasma, blastomycosis, aspergillus all came back negative. Human immunodeficiency virus (HIV) and QuantiFERON (QFT-G, Cellestis Limited, Carnegie, Vi Australia) were both negative.

On further evaluation, the patient reported living in his parent’s basement around the time of dyspnea onset but denied being exposed to mold. He moved out to go to college within a few days of symptoms onset and he has been living with three housemates since then. The patient initially denied tobacco use, recreational drug use, or being sexually active, and later admitted to binge drinking and vaping (dapping, use of nicotine, and delta-9-tetrahydrocannabinol (THC) products) once a week during social gatherings, and the last time he vaped was two days prior to the onset of dyspnea. The urine drug screen came back positive for cannabinoids.

**Figure 2:**
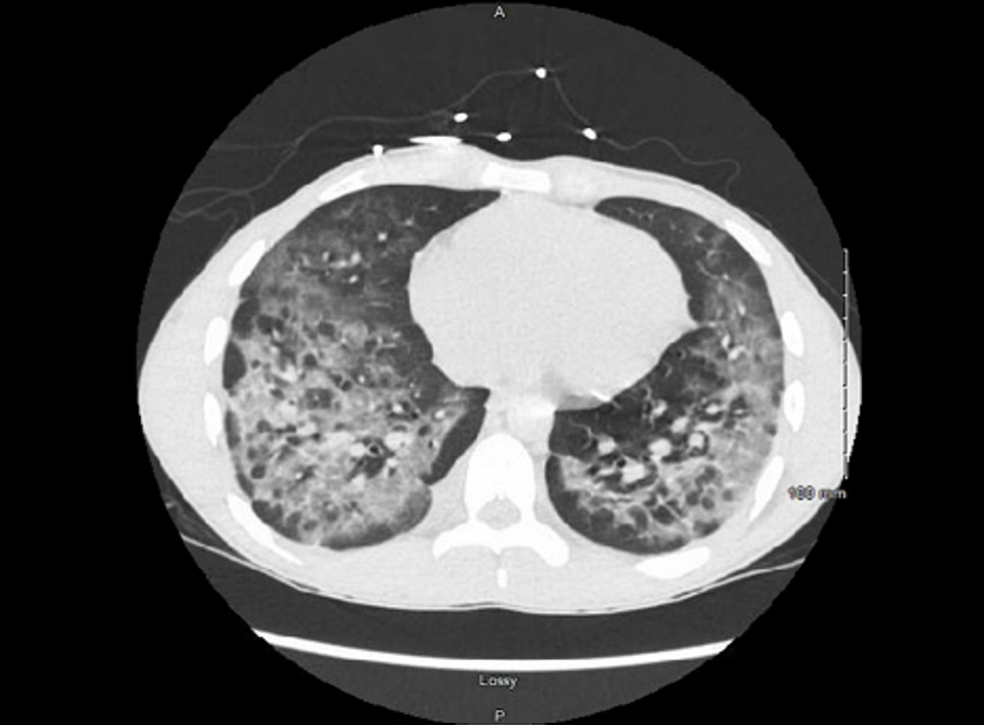
Chest CT at time of admission showing bilateral upper lobe ground-glass opacities.

Acute hypoxemic respiratory failure (AHRF) acutely worsened while on empiric therapy for CAP requiring a high flow nasal cannula (HFNC) with 35 to 45 L/min O2 and 0.4 to 0.6 FiO2, and he was started on treatment with methylprednisolone 40 mg every six hours and albuterol-ipratropium nebulizers. Infectious disease and pulmonology were consulted. Work up with CT chest demonstrated diffuse bilateral lower lobe predominant ground-glass opacities with relative sparing of the subpleural regions and associated interstitial thickening (Figure 2). Suspicion of COVID infection remained high but he had a few more negative tests. An echocardiogram showed normal size lateral ventricles and normal systolic function with an ejection fraction of 63%; left pleural effusion was noted. AHRF improved significantly to 2L of nasal cannula after two days of receiving methylprednisolone. The patient was diagnosed with EVALI as a diagnosis of exclusion and AHRF resolved with steroid therapy.

**Figure 3:**
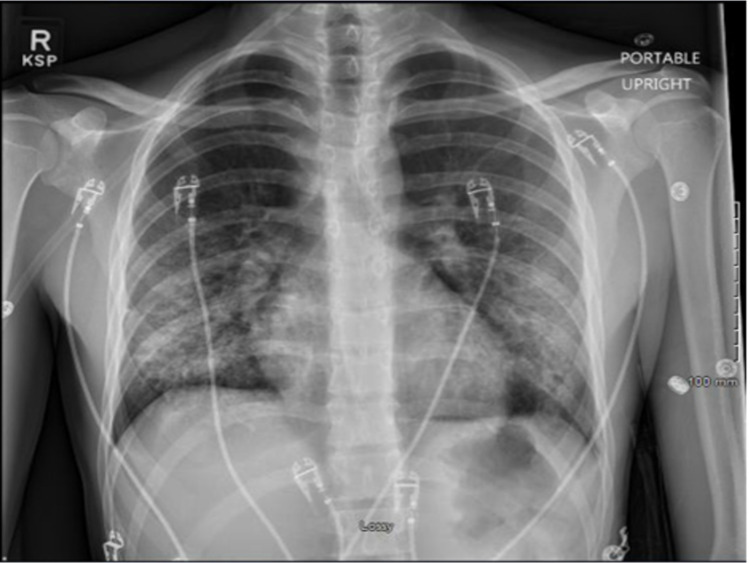
Chest X-ray after methylprednisolone treatment.

Infectious disease recommended discontinuing all antibiotics for low concern of infectious etiology. Pulmonology service recommended checking antinuclear antibodies (ANA), vasculitis panel with ANA, anti-glomerular basement membrane (anti-GBM) for initial eosinophilia. All these labs were negative, and eosinophilia was later resolved. He underwent bronchoscopy with bronchoalveolar lavage (BAL) and transbronchial biopsy. Infectious work-up on the BAL sample was unremarkable for viral, fungal, and bacterial pathogens and cytology demonstrated small clusters of atypical cells with a high nuclear to cytoplasmic ratio. The patient briefly required being on HFNC after bronchoscopy, but he was back on room air the third day after initiating methylprednisolone. Repeat CXR showed marked improvement in bilateral interstitial and soft tissue opacities (Figure 3).

The patient was hospitalized for a total of seven days, and he was discharged on prednisone 60 mg tapered dose over two weeks and supplementary oxygen (3 L of O2 with exertion). Five weeks after the hospital admission, the patient was evaluated at the pulmonary clinic for follow-up. He has been asymptomatic and AHRF has completely resolved.

## Discussion

The prevalence of vaping has significantly increased among teenagers and young adults in the US [1].­ As of February 2020, a total of 2,807 hospitalized EVALI cases or deaths have been reported to the CDC with 68 confirmed deaths [1,2]. Among these reported cases, 66% were male, and 37% of patients were 18 to 24 years old [1,2]. Many middle schools and high school students view vaping as the healthier alternative to smoking traditional cigarettes or as the first step to quitting smoking traditional cigarettes [3].

The proposed pathology explaining why vaping THC oil damages the lung is debated and poorly understood with no specific histopathological findings. This might be due to the direct impact of cannabinoid vapors or the inflammation from the diluting additives in the cartridges [4]. Most studies have shown that the most common diluting agent is vitamin-E acetate oil; harmless if used on topical application but would cause a severe inflammatory reaction in the lung parenchyma when inhaled, and it was detected in BAL samples from 48 out of 51 EVALI cases from 16 different states across the US [5-7]. The second agent, diacetyl, commonly used as a flavoring agent in foods is approved by the Food and Drug Administration (FDA) for consumption through the gastrointestinal (GI) tract, but it has been used as a flavoring agent in vape cartridges to provide a buttery flavor to the inhaled vapors and diacetyl has been shown to reduce the forced expiratory volume in one second (FEV1) [8,9]. Aerosol generation from toxic metals, such as nickel and lead, evaporation has also been studied in vaping. The burning of these metals stimulates the release of cytokines interleukin (IL)-6 and IL-8, which is a similar mechanism of injury in cigarette smoking [10,11]. Although vaping is viewed by many as harmless, these various mechanisms could lead to lung injury, and this has become a public health concern in the last few years.

In July 2019, the Wisconsin Department of Health Services and the Illinois Department of Public Health received reports of EVALI and launched a coordinated public health investigation [12]. This investigation discovered 98 cases, one out of five are male with a median age of 21 years, and the majority of these patients presented with dyspnea, cough, nausea, vomiting, diarrhea, abdominal pain, and fever [12]. These initial presenting symptoms resemble CAP or COVID-19, and thus patients are usually treated with empiric antibiotics without much improvement [1,12]. Our patient presented with dyspnea, cough, diarrhea, and fever, and was prescribed oral azithromycin for five days. His symptoms failed to improve and required him to seek medical attention after four days of being on antibiotics.

Reaching the diagnosis of EVALI could be challenging for it is a diagnosis of exclusion, and CT findings could provide some clues. Ground glass opacities are the most common presentation of EVALI, with multifocal distribution and sub-pleural sparing in about half of the patients [13]. Sub-pleural sparing was seen in our patient, and this finding is unique for sub-pleural sparing has not been noted in COVID infection, which usually presents as consolidation, crazy-paving pattern, and subpleural line opacities. Recognizing subpleural sparing patterns on imaging could point towards the diagnosis of EVALI rather than COVID infection if repeat COVID testing and BAL sample from bronchoscopy are both negative [14].

There are no randomized controlled trials that have evaluated therapy for EVALI [15]. However, there is some evidence that the use of high-dose glucocorticoids (60 mg) may be helpful in decreasing the inflammatory response in EVALI [15-17]. Due to the substantial risk of acute respiratory distress syndrome (ARDS) and rapid decline in respiratory failure, it is important to start corticosteroid treatment early in severely ill patients [17]. The initial recommended dosage that has been studied in literature is 60 mg. Corticosteroids should be tapered with clinical improvement, usually, patients do not need more than two weeks of therapy [17]. The long-term effects of EVALI are unknown [16]. The prognosis tends to be good for patients, but there have been fatalities described [17]. Many patients have no long-term effects and improve within a week of presentation after six months of follow-up [16]. Some patients require oxygen therapy for persistent hypoxia and prolonged pulmonary follow-up [15]. Fifty percent of patients who have relapsing symptoms which require readmission have resumed e-cigarette consumption [17]. All patients with EVALI should also be counseled for cessation of use of e-cigarettes and tobacco cigarettes, as cessation may decrease symptoms and recurrence of disease [15].

## Conclusions

As the prevalence of EVALI continues to rise, clinicians should have a high suspicion of EVALI in patients who present with hypoxemia and negative infectious workup. Administration of corticosteroids has shown remarkable efficacy in improving hypoxemia; however, many patients may have chronic lung injury and may require oxygen long term. Cases of EVALI should continue to be reported in order to better monitor the trend of the disease.
